# Systemic lupus erythematosus with various clinical manifestations in a patient with hereditary angioedema: a case report

**DOI:** 10.1186/s13223-022-00725-8

**Published:** 2022-09-18

**Authors:** Yusuke Ushio, Risa Wakiya, Tomohiro Kameda, Shusaku Nakashima, Hiromi Shimada, Mai Mahmoud Fahmy Mansour, Mikiya Kato, Taichi Miyagi, Koichi Sugihara, Rina Mino, Mao Mizusaki, Emi Ibuki, Norimitsu Kadowaki, Hiroaki Dobashi

**Affiliations:** 1 Division of Hematology, Rheumatology and Respiratory Medicine, Department of Internal Medicine, Faculty of Medicine, Kagawa University, 1750-1 Ikenobe, Miki-cho, Kita-gun, Kagawa, 761-0793 Japan; 2 Department of Diagnostic Pathology, Faculty of Medicine, Kagawa University, Kagawa, Japan

**Keywords:** Hereditary angioedema, C1 esterase inhibitor, Systemic lupus erythematosus, Lupus nephritis

## Abstract

**Background:**

Hereditary angioedema (HAE) is an inherited disease characterized by recurrent angioedema without urticaria or pruritus. The most common types of HAE are caused by deficiency or dysfunction in C1 esterase inhibitor (C1-INH-HAE). The association between C1-INH-HAE and systemic lupus erythematosus (SLE) is known; however, variations in the underlying pathophysiology, disease course, and treatment in this population remain incompletely understood.

**Case presentation:**

A 31-year-old Japanese woman with a prior diagnosis of HAE type 1 based on the episodes of recurrent angioedema, low C1 inhibitor antigen levels and function, and family history presented with new complaints of malar rash, alopecia, and arthralgias in her hands and elbows. She later developed fever, oral ulcers, lupus retinopathy, a discoid rash localized to her chest, and malar rash. Investigations revealed positive antinuclear antibody, leukopenia, thrombocytopenia, hypocomplementemia, and nephritis. Based on these findings, she was diagnosed with SLE according to the 2019 European League Against Rheumatism/American College of Rheumatology classification criteria. There did not appear to be a correlation between HAE disease activity and the timing of presentation with SLE, because HAE disease activity had been stable. The patient was able to achieve and maintain remission with immunosuppressive therapy including prednisolone, hydroxychloroquine, and tacrolimus.

**Conclusions:**

Our patient presented with a variety of symptoms, including fever and cytopenia in addition to mucocutaneous, joint, ocular, and renal lesions. It is important to better characterize the clinical characteristics of SLE in patients with C1-INH-HAE, and to clarify the mechanisms of SLE in this population.

## Background

Hereditary angioedema (HAE) is an inherited disease characterized by recurrent angioedema without urticaria or pruritus [1]. This disease was first described by Osler in 1888 [2]. The prevalence in Europe and the United States is estimated to be about 1 in 50,000, with no racial differences [3].

There are three types of HAE, two of which are well recognized. HAE type 1 occurs in approximately 85% of patients and is characterized by decreased production of C1 esterase inhibitor (C1-INH), reducing the functional activity to 5–30% of normal, whereas HAE type 2 occurs in approximately 15% of patients and is characterized by normal or elevated antigen levels of C1-INH but reduced functional activity. Abnormalities in the SERPING1 gene are the cause of the reduced levels and function of C1-INH. HAE types 1 and 2 are referred to as C1-INH-HAE. However, a third group of patients with HAE has been described. These patients have a clinical presentation similar to that of C1-INH-HAE but with normal C1-INH antigen levels and function. This new type of HAE is classified as HAE with normal C1-INH (nC1-INH-HAE). This category is a mixed group with likely multiple underlying genetic causes, including factor XII, angiopoietin-1, plasminogen, kininogen-1, and myoferlin without mutation in the SERPING1 gene [1, 4]. In C1-INH-HAE, bradykinin is overproduced owing to activation of the plasma contact system, which increases vascular permeability and causes repeated localized edema in various parts of the body, including the skin, gastrointestinal tract, and respiratory tract. The edema resolves spontaneously and disappears in 2–5 days without treatment; however, laryngeal lesion can cause fatal asphyxia [5].

An association of C1-INH-HAE with autoimmune diseases was previously reported [6–17]. Systemic lupus erythematosus (SLE) is well reported in patients with C1-INH-HAE, and several cases have been reported [6, 12, 13, 15–17]. However, information on the underlying pathophysiology, clinical manifestations, disease course, and response to treatment in this population is limited. In particular, it remains unclear whether SLE in this population is distinct from SLE in patients without HAE and whether there may be similarities to SLE seen in patients with complement deficiencies. This case report presents a patient with C1-INH-HAE who develops SLE with various clinical manifestations.

## Case presentation

A 31-year-old Japanese woman had a history of swelling of the lips without any specific triggers, which she began experiencing at the age of 14 years. She also had a family history of HAE; her mother and grandmother were diagnosed with HAE. At the age of 20 years, she was diagnosed with HAE type 1 based on blood test results showing low C4 and C1-INH antigen levels and function, in addition to a family history of HAE. The patient has had episodes of skin and gastrointestinal angioedema several times per year, as well as one occurrence of laryngeal edema. For these episodes she was treated with plasma-derived C1-INH (Berinert [CSL Behring, Marburg, Germany]) at a hospital near her home. She was not treated prophylactically at her own preference.

At the age of 30 years, she developed malar rash, alopecia, and arthralgia in her hands and elbows. At the onset of these symptoms, the patient experienced HAE attacks of the skin and gastrointestinal tract approximately twice per year. The arthralgia was considered to be arthritis associated with Sjögren’s syndrome, which was suspected based on blood test results showing a high level of serum anti-Sjögren’s-syndrome-related antigen A (anti-SS-A) antibody (> 1200 U/mL; normal, < 10 U/mL). She was prescribed prednisolone up to 10 mg/day.

At a follow-up 8 months later, she reported general malaise. Her temperature was > 39 °C. Physical examination showed oral ulcers and discoid rash on the anterior chest, and she was admitted to the local hospital. Antibiotics were started, but her symptoms did not improve. SLE was suspected based on the clinical symptoms, and an increased dose of prednisolone (20 mg/day) was initiated.

**Fig. 1:**
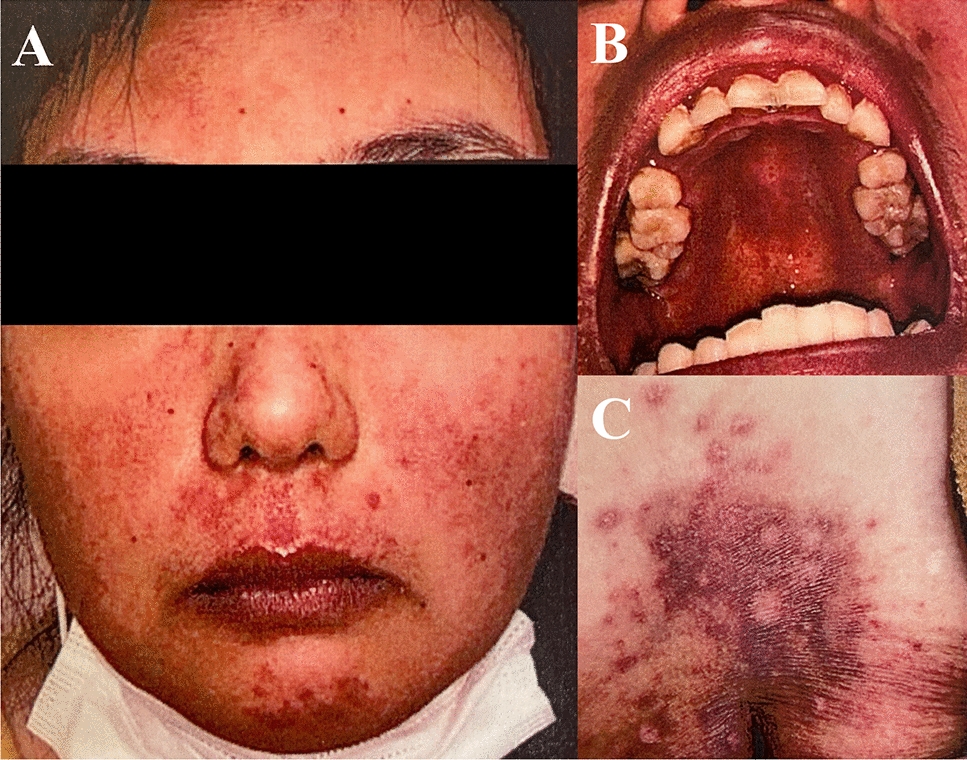
Cutaneous mucosal lesions before treatment: **A** malar rash; **B** oral ulcers; and **C** discoid rash

**Fig. 2:**
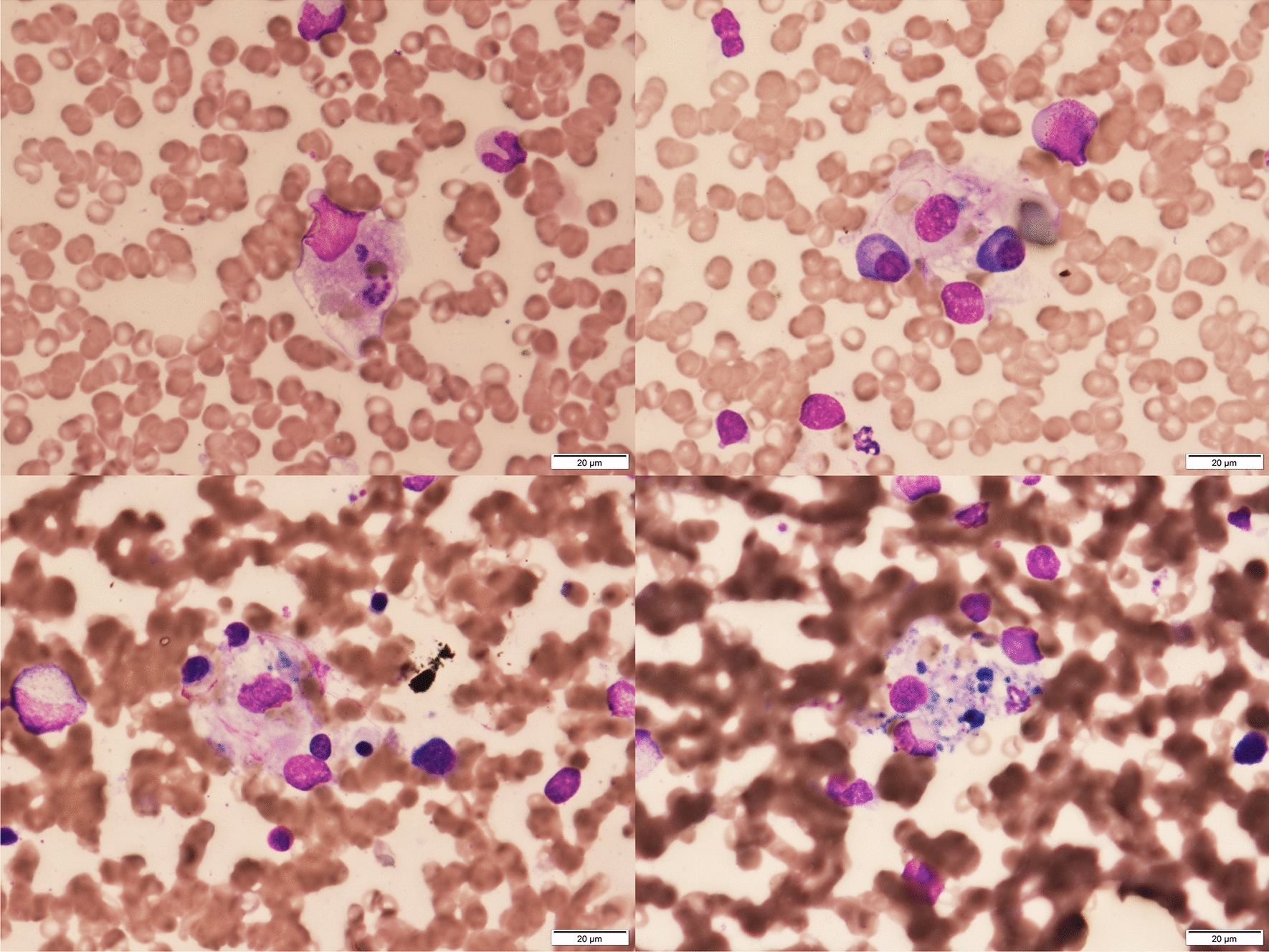
Bone marrow aspirate (May-Grunwald Giemsa stain, × 400) shows hemophagocytosis by macrophages

**Fig. 3:**
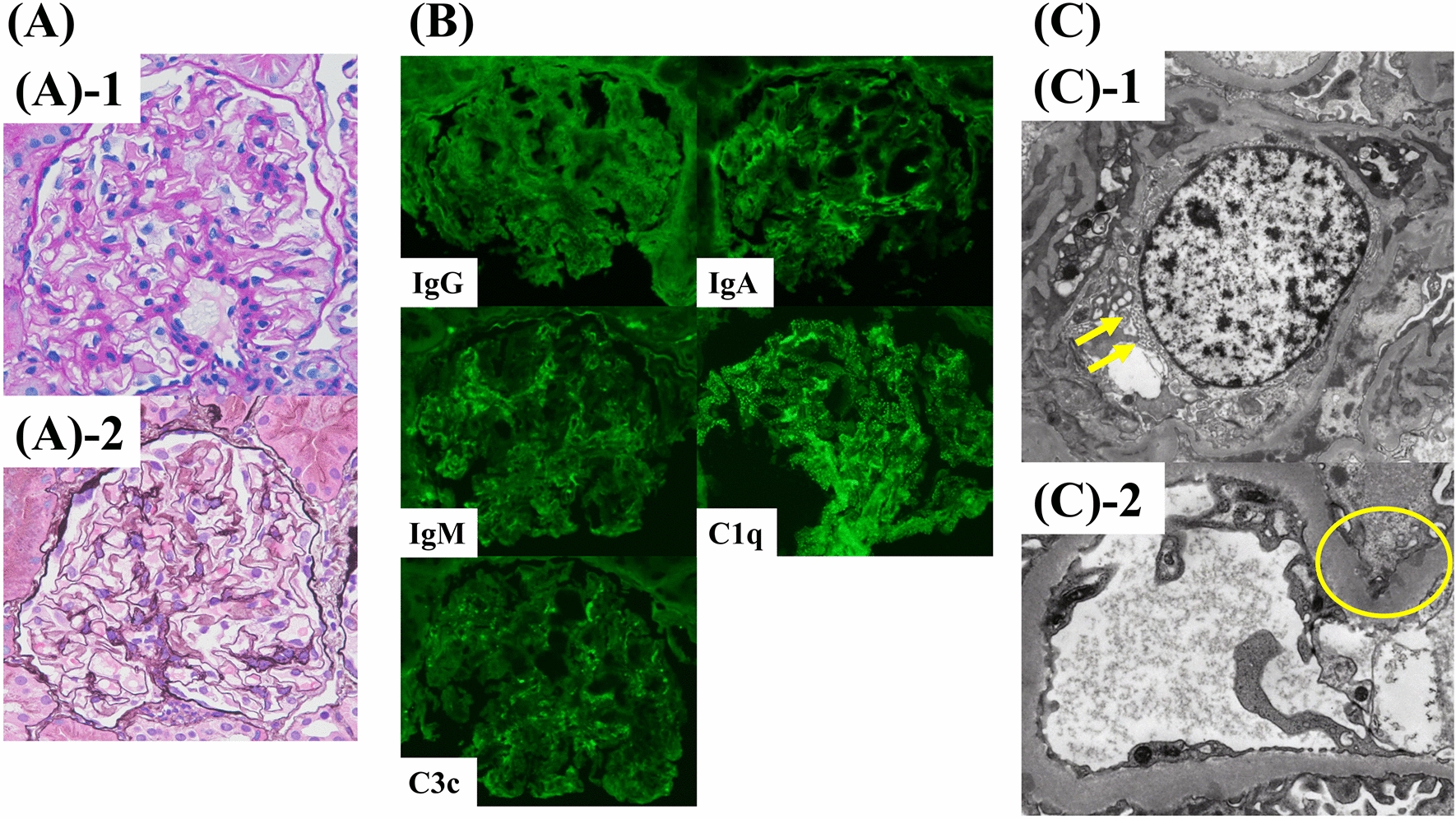
Renal biopsy. **A** Light microscopy: A-1) periodic acid–Schiff stain; and A-2) periodic acid–methenamine–silver stain. No mesangial proliferation, double contour of the glomerular basement membranes, or irregular spike formations are noted. **B** Immunofluorescence study shows deposition of immunoglobulin (Ig) A, IgM, and complement **C** 1q and C3c in glomeruli. **C** Electron microscopy: C-1) tubuloreticular inclusions (yellow arrow) were seen in some endothelial cells; and C-2) subepithelial electron-dense deposits (yellow circle) were observed

Four days later, she was transferred to our hospital for further diagnosis and specialized treatment. On physical examination, malar rash, oral ulcers, discoid rash, and arthritis in her elbows and knees were observed (Fig. 1). Urinalysis showed proteinuria and occult blood. Laboratory data around the time of presentation are shown in Table 1 and are significant for the following elevated values: C-reactive protein (CRP), 5.65 mg/dL (normal, < 0.20 mg/dL); aspirate transaminase (AST), 85 U/L (normal, < 30 U/L); and alanine transaminase (ALT), 154 U/L (normal, < 23 U/L). Results also showed leukopenia with a white blood cell (WBC) count of 1140/μL (normal, 4700–8700/μL; neutrophils, 809/μL) and thrombocytopenia with a platelet count of 9.7 × 10^4^/μL (normal, 15.0–35.0 × 10^4^/μL), elevated lactate dehydrogenase at 537 U/L (normal, 124–222 U/L), and ferritin at 622 ng/mL (normal, 6–138 ng/mL), in addition to low C4 and CH50 levels: complement C3, 111 mg/dL (normal, 68–144 mg/dL); complement C4, < 2 mg/dL (normal, 12–33 mg/dL); and complement, total (CH50), < 14.0 U/mL (normal, 30–46 U/mL). Assessment of autoantibodies was positive for antinuclear antibody (ANA) (1:80, nucleolar pattern, speckled pattern), anti-ribonucleoprotein antibody (14.2 U/mL; normal, < 10 U/mL), and anti-SS-A autoantibody (48,550 U/mL; normal, < 10 U/mL). Anti-double-stranded-DNA (anti-ds-DNA) antibody (immunoglobulin G) and anti-Smith antibody were negative. Bone marrow examination was performed to find the cause of cytopenia. Results showed no proliferation of blasts or tumor cells, but hemophagocytosis by macrophages was observed (Fig. 2). Computed tomography showed obvious splenomegaly. A thorough examination of the organ lesions revealed lupus retinopathy and lupus nephritis. Based on the pathological findings, a pathologist (E.I.) confirmed the diagnosis of lupus nephritis class I according to the International Society of Nephrology/Renal Pathology Society classification (Fig. 3) [18]. Based on the findings of positive ANA, fever, nonscarring alopecia, oral ulcers, malar rash, discoid rash, arthralgia, leukopenia, thrombocytopenia, hypocomplementemia, lupus retinopathy, and nephritis, she was diagnosed with SLE according to the 2019 European League Against Rheumatism/American College of Rheumatology classification criteria [19].

**Table 1:** Laboratory data around the time of presentation

Blood test					
		Normal range			Normal range
WBC, /μL	1140	4700–8700	anti-nuclear Ab	1: 80	< 40
Stab, %	7.0	0.0–13.0	(staining patterns)	nucleolar, speckled	
Seg, %	64.0	38.0–58.9	anti-ds-DNA Ab, IU/mL	< 5.0	0.0–12.0
Eos, %	0.0	0.2–6.8	anti-Smith Ab, U/mL	< 3.0	0.0–10.0
Baso, %	1.0	0.0–1.0	anti-RNP Ab, U/mL	14.2	0.0–10.0
Mono, %	8.0	2.3–7.7	anti-SS-A Ab, U/mL	48,550.0	0.0–10.0
Lym, %	19.0	26.0–46.6	anti-SS-B Ab, U/mL	< 3.0	0.0–10.0
RBC, × 10^4/μL	456	370–490	Direct Coombs	( +)	
Hb, g/dL	12.5	11.0–15.0	Indirect Coombs	(-)	
Hct, %	37.2	35.0–45.0	anti-platelet Ab	(-)	
Plt, × 10^4/μL	9.7	15.0–35.0	PA-IgG, ng/10^7^cells	42.7	< 46
			Lac, ratio	1.13	0.00–1.30
CRP, mg/dL	1.78	0.00–0.20	CL-IgG, U/mL	< 8.0	0.0–10.0
TP, g/dL	7.6	6.5–8.2	CLβ2-GP1 Ab, U/mL	< 0.7	0.0–3.5
Alb, g/dL	3.2	3.5–5.5			
BUN, mg/dL	6.1	7.0–20.0	β-D glucan, pg/mL	< 6.0	0.0–11.0
Cre, mg/dL	0.37	0.50–1.00	Procalcitonin, ng/mL	0.05	0.00–0.49
GOT, IU/L	85	13–30	HBs-Ag	(-)	
GPT, IU/L	154	7–23	HCV-Ab	(-)	
ALP, IU/L	108	106–322	EBV-VCA-IgG	( +)	
γ-GTP, IU/L	29	9–32	EBV-VCA-IgM	(-)	
LDH, IU/L	537	124–222	EBV-EA-IgG	(-)	
Amylase, U/L	51	44–132	EBV-EA-IgM	(-)	
Ferritin, ng/mL	622	6.0–138	EBNA-Ab	( +)	
sIL-2R, U/mL	855	121–613	CMV-IgG	( +)	
IgA, mg/dL	570	114–435	CMV-IgM	(-)	
IgG, mg/dL	2894	870–1700	CMV Ag (10, 11)	(-)	
IgM, mg/dL	87	46–260	Parvovirus B19-IgM	(-)	
C3, mg/dL	111	68–114			
C4, mg/dL	< 2	12–33	Urinalysis		
CH50, U/mL	< 14	30–50	Protein	( ±)	
C1 inhibitor activity, %	< 25	70–130	Protein, g/gCr	0.21	
			RBC	(1 +)	
C1 inhibitor levels, mg/dL	< 3	21–39	NAG, U/L	15.5	0.7–11.2
			β2-microglobulin, µg/L	3259	0–250
PT-INR	0.93	0.85–1.15			
APTT, sec	26.4	27.0–47.0			
Fibrinogen, mg/dL	329	200–400			
D-dimer, μg/mL	3.7	0.0–1.0			

*sIL-2R* soluble interleukin-2 receptor, *anti-ds-DNA Ab* anti-double-stranded-DNA antibody, *anti-RNP Ab* anti-ribonucleoprotein antibody, *PA-IgG* platelet-associated immunoglobulin G, *CL-IgG* anti-cardiolipin immunoglobulin G, *CLβ2-GP1 Ab* anti-cardiolipin-beta2-glycoprotein I complex antibody, *NAG* N-acetyl-beta-glucosaminidase

**Fig. 4:**
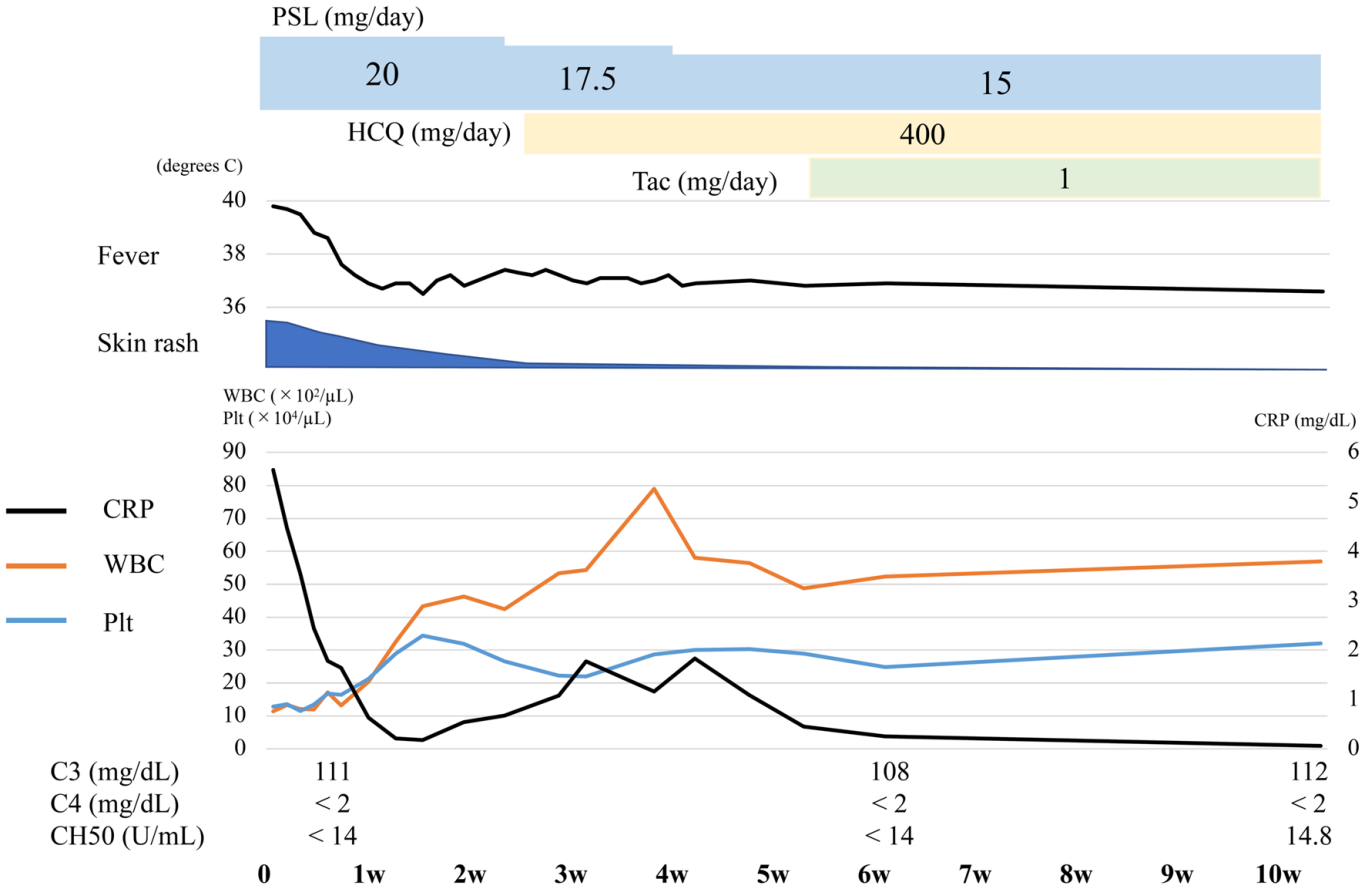
Clinical course of treatment, symptoms, and laboratory data

**Fig. 5:**
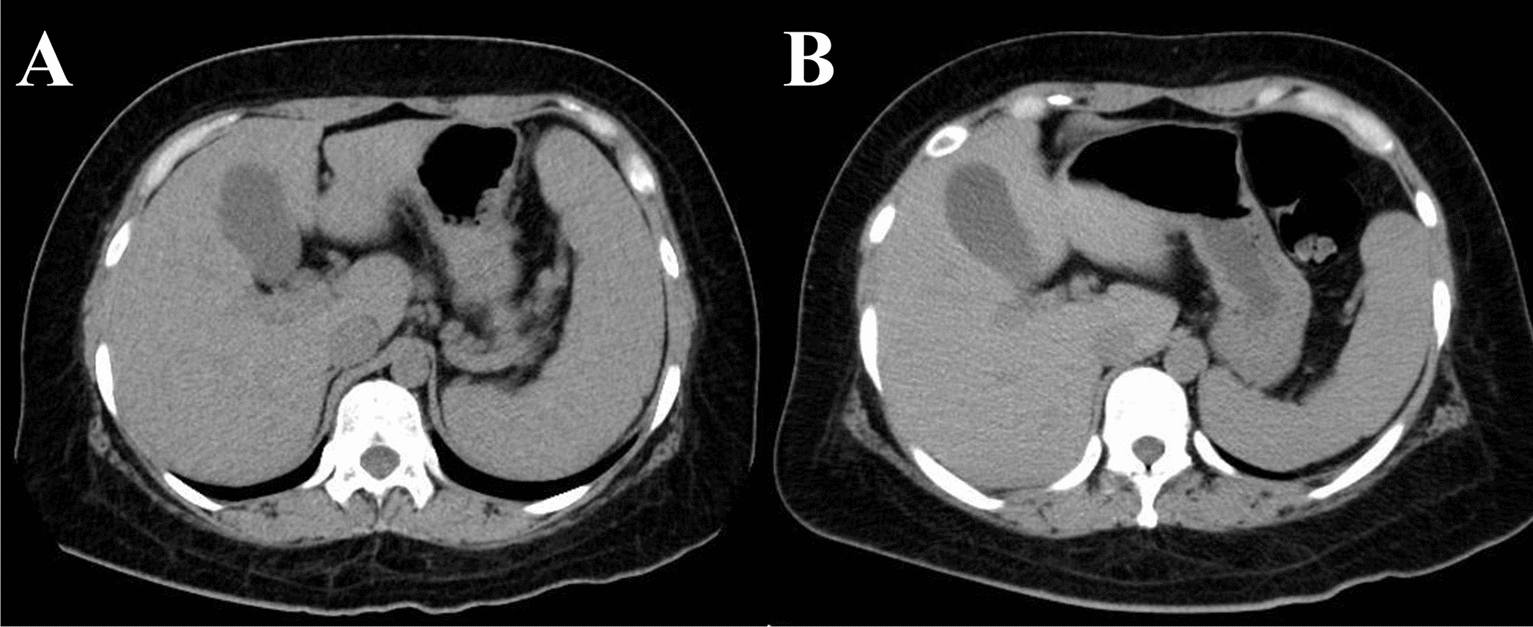
Computed tomography: **A** on admission to the authors’ hospital; and **B** 3 weeks after treatment initiation. The patient had significant splenomegaly on admission, which improved after the treatment

After starting treatment with 20 mg/day prednisolone, the clinical symptoms improved, so prednisolone was tapered with the concomitant use of 400 mg/day hydroxychloroquine and 1 mg/day tacrolimus. After 4 weeks, her oral ulcers, malar rash, discoid rash, and arthralgia had disappeared and WBCs and platelets had increased (Fig. 4). Her splenomegaly had also improved (Fig. 5).

At a follow-up examination 7 months later, it was noted that the patient was able to maintain remission of SLE by continuing 7 mg/day prednisolone, 400 mg/day hydroxychloroquine, and 2 mg/day tacrolimus. Her hypocomplementemia has improved slightly but persisted (C3, 113 mg/dL; C4, 4 mg/dL; CH50, 20.1 U/mL), which is thought to be associated with HAE. The patient continues to experience episodes of HAE several times per year, but the frequency of these episodes has not worsened since before she started treatment for SLE.

## Discussion and conclusions

The literature includes several reports of concomitant HAE and autoimmune diseases [6–17]. The rates of autoimmune disease in patients with HAE are estimated to range from 11 to 12% [6, 11] compared with a baseline prevalence of 4.5% in the general population [20]. SLE is one of the most common autoimmune diseases in patients with C1-INH-HAE. In 2020, Levy et al. reviewed cases of concomitant C1-INH-HAE and autoimmune diseases [16]. Of 155 cases, lupus or lupus-like diseases accounted for 52 cases (34%) and SLE accounted for 30 cases (19%).

The current case presentation describes a patient with C1-INH-HAE who subsequently developed SLE. Although genetic testing for SERPING1 was not available due to lack of patient consent, the diagnosis of C1-INH-HAE was very likely based on low C1-INH levels and function as well as the patient’s family history. However, genetic testing for the SERPING1 gene mutation is essential to obtain a genetic diagnosis of C1-INH-HAE, and the familial diagnosis should be confirmed with genetic testing and subsequent allele segregation of symptomatic and asymptomatic individuals. In addition, since anti-C1-INH antibodies were not measured, acquired angioedema in SLE cannot be completely ruled out; it has been reported that high anti-C1-INH IgG levels were found in 17% of SLE patients (only 4% of controls; *p* = 0.0003) and in some patients presenting with angioedema symptoms [21].

In patients with C1-INH-HAE, findings of low C4 and low CH50 levels are common due to chronic C1 activation with secondary consumption of C4 and other early complement components, and there is thought to be an association between disease status and these levels [22]. Other causes of low C4 and CH50 levels include a deficiency of other early complement components. Deficiencies of early complement are also associated with SLE. Although we did not complete the genetic testing for congenital complement deficiencies, we felt that these diagnoses were less likely because the patient had no history of severe recurrent pyogenic infections in early life but had a history of recurrent angioedema and low C1-INH levels. Low C4 and CH50 levels are also common in patients with active SLE. Given our patient’s multiple SLE manifestations at the time of presentation, even in the absence of the HAE diagnosis, these values would be expected to be low. In this case, after we initiated treatment for SLE and the patient’s active SLE symptoms resolved, the C4 and CH50 levels were still low but slightly increased compared to previous values. Active SLE may have contributed to the lower values at the time of SLE diagnosis.

There are few reports on the patient characteristics and clinical manifestations in patients with C1-INH-HAE who develop SLE. In 2015, Sérézal et al. performed a literature review of 32 patients with C1-INH-HAE who developed lupus [15]. The median age at HAE onset was 14 years (3–30 years); the median age at lupus onset was 19.5 years (1–78 years). Of the 32 study patients, 15 (47%) had SLE, 6 (19%) had subacute lupus, 8 (25%) had discoid lupus, and 3 (9%) had lupus-like syndrome or an unspecified cutaneous lupus. Of the 15 patients diagnosed with SLE, 8 (53%) had mucocutaneous lesions, which a rate is higher than that reported in a European study (31.6%) [23]. These findings suggest that cutaneous findings may be more common in lupus associated with C1-INH-HAE. In addition, renal lesions were found in eight patients in this study [15]. Most cases are reported to be as mild, similar to the patient in this study, whereas Khan et al. reported a case of lupus nephritis that required intensive immunosuppression therapy [10]. In terms of immunologic abnormalities, in the same study, ANA was detected in 20 of the 31 patients (64.5%), anti-ds-DNA antibodies in 2 of the 17 patients (11.8%), and anti-SS-A antibodies in 5 of the 9 patients (55.6%), suggesting a relatively high prevalence of ANA and anti-SS-A antibodies and a low prevalence of anti-ds-DNA antibodies in lupus associated with C1-INH-HAE. Similarly, in our patient, ANA and anti-SS-A antibodies were positive; however, anti-ds-DNA antibodies were negative.

Based on the findings of fever, splenomegaly, cytopenia, elevated ferritin, and hemophagocytosis on bone marrow biopsy, our patient met the criteria for hemophagocytic lymphohistiocytosis (HLH) [24]. This is relatively uncommon in SLE, occurring in only 0.9%–4.6% of cases [25]. To our knowledge, there are no existing case reports of HLH secondary to SLE in patients with underlying HAE.

The pathogenesis of SLE in patients with C1-INH-HAE currently remains unclear; however, a decrease in C4 and other early complement components may be associated with an increased risk for SLE [26]. One of the main functions of C1-INH is to regulate the complement pathway by preventing excessive activation of C4 and C2 through inhibition of the complement protease C1 in the classical pathway and mannose-binding lectin-associated serine protease 1/2 (MASP1/2) in the lectin pathway [27]. In contrast, the deficient function of C1-INH in HAE patients leads to autoactivation of C1, resulting in chronic activation and consumption of C4 and other early complement components.

The complement system plays a protective role in developing autoimmune diseases by contributing to clearance of immune complexes, clearance of apoptotic cells that may be the source of autoantigens, and tolerance to self-antigens [28–30]. Genetic deficiencies in C1q, C4, and C2 are thought to increase the risk of developing the autoimmune diseases such as SLE and glomerulonephritis by lack of these protective mechanisms [30, 31].

SLE develops in 75% of cases of congenital C4 deficiency. The clinical features of SLE secondary to C4 deficiency are early onset, cutaneous manifestations, and mild renal lesions [32]. The positivity rates for ANA and anti-SS-A antibodies are 75% and 70%, respectively, whereas the positivity rate for anti-ds-DNA antibodies is as low as 18% [33]. These features are similar to those of our case and previous cases of SLE associated with C1-INH-HAE [10, 13, 15]. Significantly low levels of C4 related to hypercatabolism are observed in C1-INH-HAE, which might explain the similarities between SLE secondary to C4 deficiency and SLE associated with C1-INH-HAE. In congenital homozygous C2 deficiency, SLE occurs in 10% of cases, and similarities in clinical manifestations between SLE secondary to C2 deficiency and SLE associated with C1-INH-HAE have also been reported: few renal lesions, low positivity rate for anti-ds-DNA antibodies, absent lupus bands, and frequent discoid lupus [15]. In congenital C1q deficiency, SLE occurs in approximately 90% of cases. In SLE secondary to C1q deficiency, skin lesions are present in 90% of cases, with relatively high positivity for ANA and anti-SS-A antibodies (75% and 70%, respectively) but low positivity for anti-ds-DNA antibodies (20%) [33], which suggests similarities with the features of SLE associated with C1-INH-HAE.

It is worthwhile to examine whether the prophylactic administration of C1-INH in patients with HAE improves the decrease in complement components associated with C1-INH-HAE, leading to an improvement in SLE symptoms. It has been reported that the prophylactic administration of C1-INH in patients with HAE normalizes C4 and C1-INH antigen and functional levels [34, 35]. However, whether C1-INH prophylaxis affects other complement components or improves the clinical manifestations of autoimmune diseases in patients with C1-INH-HAE remains unclear. The efficacy of prophylactic therapy with the normalization of C4 levels needs investigation in the future. In addition, examining HAE disease activity/C4 levels as a potential modifying factor for the development of SLE symptoms would be interesting. In this case, there did not appear to be a correlation between HAE disease activity and the timing of presentation with SLE, because HAE disease activity had been stable without the need for prophylactic therapy.

The current case presentation describes a patient with C1-INH-HAE with a subsequent diagnosis of SLE and HLH. Registries for patients with HAE are needed to more accurately identify the frequency of SLE in this population, to better characterize the clinical characteristics of SLE in this population, and to determine whether HAE disease activity and management could play a role in SLE presentation. In addition, further studies are required to determine whether SLE associated with C1-INH-HAE is similar to SLE associated with complement deficiencies and whether it is distinct from SLE unrelated to complement deficiencies or HAE.

## Data Availability

The dataset supporting the conclusions of this article is available upon reasonable request from the corresponding author.

## Abbreviations

ANA: Antinuclear antibody
anti-SS-A: Anti-Sjögren’s-syndrome-related antigen A
ALT: Alanine transaminase
AST: Aspirate transaminase
CRP: C-reactive protein
C1-INH: C1 esterase inhibitor
HAE: Hereditary angioedema
SLE: Systemic lupus erythematosus
WBC: White blood cell
